# Surveillance of SARS-CoV-2 in Frankfurt am Main from October to December 2020 Reveals High Viral Diversity Including Spike Mutation N501Y in B.1.1.70 and B.1.1.7

**DOI:** 10.3390/microorganisms9040748

**Published:** 2021-04-02

**Authors:** Marek Widera, Barbara Mühlemann, Victor M. Corman, Tuna Toptan, Jörn Beheim-Schwarzbach, Niko Kohmer, Julia Schneider, Annemarie Berger, Talitha Veith, Christiane Pallas, Tobias Bleicker, Udo Goetsch, Julia Tesch, Rene Gottschalk, Terry C. Jones, Sandra Ciesek, Christian Drosten

**Affiliations:** 1 Institute for Medical Virology, University Hospital Frankfurt, Goethe University Frankfurt am Main, 60596 Frankfurt am Main, Germany; Tuna.ToptanGrabmair@kgu.de (T.T.); Niko.Kohmer@kgu.de (N.K.); annemarie.berger@em.uni-frankfurt.de (A.B.); christiane.pallas@kgu.de (C.P.); Sandra.ciesek@kgu.de (S.C.); 2 German Centre for Infection Research (DZIF), Institute of Virology, Charité—Universitätsmedizin Berlin, Humboldt—Universität zu Berlin, 10117 Berlin, Germany; barbara.muehlemann@charite.de (B.M.); victor.corman@charite.de (V.M.C.); joern.beheim-schwarzbach@charite.de (J.B.-S.); julia.schneider@charite.de (J.S.); Talitha.Veith@charite.de (T.V.); Tobias.Bleicker@charite.de (T.B.); julia.tesch@charite.de (J.T.); terry@jon.es (T.C.J.); christian.drosten@charite.de (C.D.); 3 Public Health Department of the City of Frankfurt am Main, 60313 Frankfurt am Main, Germany; udo.goetsch@stadt-frankfurt.de (U.G.); rene.gottschalk@stadt-frankfurt.de (R.G.); 4 Centre for Pathogen Evolution, Department of Zoology, University of Cambridge, Downing St., Cambridge CB2 3EJ, UK; 5 German Center for Infection Research, DZIF, 60596 Braunschweig, Germany; 6 Fraunhofer Institute for Molecular Biology and Applied Ecology (IME), Branch Translational Medicine and Pharmacology, 60596 Frankfurt am Main, Germany

**Keywords:** SARS-CoV-2, genetic diversity, molecular surveillance, B.1.1.7, natural selection, spike mutation, N501Y

## Abstract

Background: International travel is a major driver of the introduction and spread of SARS-CoV-2. Aim: To investigate SARS-CoV-2 genetic diversity in the region of a major transport hub in Germany, we characterized the viral sequence diversity of the SARS-CoV-2 variants circulating in Frankfurt am Main, the city with the largest airport in Germany, from the end of October to the end of December 2020. Methods: In total, we recovered 136 SARS-CoV-2 genomes from nasopharyngeal swab samples. We isolated 104 isolates that were grown in cell culture and RNA from the recovered viruses and subjected them to full-genome sequence analysis. In addition, 32 nasopharyngeal swab samples were directly sequenced. Results and conclusion: We found 28 different lineages of SARS-CoV-2 circulating during the study period, including the variant of concern B.1.1.7 (Δ69/70, N501Y). Six of the lineages had not previously been observed in Germany. We detected the spike protein (S) deletion Δ69/Δ70 in 15% of all sequences, a four base pair (bp) deletion (in 2.9% of sequences) and a single bp deletion (in 0.7% of sequences) in ORF3a, leading to ORF3a truncations. In four sequences (2.9%), an amino acid deletion at position 210 in S was identified. In a single sample (0.7%), both a 9 bp deletion in ORF1ab and a 7 bp deletion in ORF7a were identified. One sequence in lineage B.1.1.70 had an N501Y substitution while lacking the Δ69/70 in S. The high diversity of sequences observed over two months in Frankfurt am Main highlights the persisting need for continuous SARS-CoV-2 surveillance using full-genome sequencing, particularly in cities with international airport connections.

## 1. Introduction

Severe acute respiratory syndrome coronavirus 2 (SARS-CoV-2), the causative agent of Coronavirus disease 2019 (COVID-19), was initially detected in the city of Wuhan, China, in December 2019. Since its introduction in the human population, the virus has diversified into a number of genetic lineages, characterized by specific amino acid substitutions. A number of substitutions have been linked to functional changes. In particular, the D614G substitution in the Spike (S) protein, which arose early in the pandemic, is associated with higher transmissibility [1]. Viruses with D614G substitution are now the dominant circulating lineages. A number of substitutions in S have been associated with immune escape (e.g., E484K, K417N) [2], or increased binding to the ACE-2 receptor (e.g., N501Y) [3]. Recently, three variants have emerged (“variants of concern”), which are associated with higher transmissibility and/or mutations allowing the escape from pre-existing immunity. These variants are referred to as B.1.1.7, B.1.351, and P.1, and were first observed in the United Kingdom, South Africa, and Brazil, respectively. Lineage B.1.1.7 has seventeen lineage-defining mutations including a deletion at positions 69/70 (Δ69/70) and 144, and substitutions N501Y and P681H in S. The N501Y substitution in S, either alone or in combination with other mutations, was suggested to have an increased binding affinity to the human receptor ACE2, and B.1.1.7 was predicted to be more transmissible [4]. This prediction was mainly supported by epidemiological studies [5,6]. Furthermore, Δ69/70 has been associated with increased infectivity in cell culture [7]. In addition, B.1.1.7 was approximately 2-fold less sensitive to neutralization by sera from convalescent individuals and recipients of two different vaccines—mRNA-1273 (Moderna) and the protein nanoparticle NVX-CoV2373 (Novavax) vaccines [8]. The neutralization of B.1.1.7 S corresponding pseudoviruses was not affected by BNT162b2 (BioNTech/Pfizer) vaccine-elicited human sera [9], but studies using cDNA-based SARS-CoV-2 clones with spike Δ69/70, E484K and N501Y revealed moderately diminished protection [10]. These early studies show that variants of concern such as B.1.1.7 might have increased infectivity, evade natural and vaccine induced immunity, and thus require continuous observation.

International travel has been highlighted as a major driver for the introduction of SARS-CoV-2 variants into areas without previous circulation [11]. Frankfurt am Main, situated in the state of Hesse, is the fifth-largest city in Germany, with a population of approximately 763,000. Due to its connection to Frankfurt Airport, the 14th largest airport in the world, serving more than 70 million passengers in 2019 (Luftverkehrsstatistik 2019, Fraport AG), Frankfurt represents a central hub for international passenger traffic. Thus, in this study we retrospectively sequenced 136 samples collected in Frankfurt am Main from October to December 2020, allowing us to describe the circulating diversity of SARS-CoV-2 lineages in the city and the introduction of variants of concern early in the second wave of infections in Germany.

## 2. Materials and Methods

### 2.1. Sample Preparation and RT-qPCR-Testing

All SARS-CoV-2 isolates were obtained from nasopharyngeal swabs of patients with suspected SARS-CoV-2 infection screened by the Public Health Department of the City of Frankfurt am Main, Germany. Swab material was suspended in 1.5 mL PBS and split for RT-qPCR-testing and viral outgrowth assay. For RT-qPCR analysis, 500 µL of the swab dilution was mixed with lysis buffer (1:1 ratio) and subjected to RT-qPCR-analysis using the SARS-CoV-2 Test on the cobas 6800 system (Roche Diagnostics International AG, Rotkreuz, Switzerland) according to the manufacturer’s protocol. Master mix was supplemented with an internal RNA control and primer-probe sets targeting SARS-CoV-2 ORF1ab and E-gene as described by the manufacturer.

### 2.2. Cell Culture and Viral Outgrowth Assay

Caco-2 (human colon carcinoma) cells were cultured in Minimum Essential Medium (MEM) supplemented with 10% fetal calf serum (FCS), 100 IU/mL of penicillin, and 100 g/mL of streptomycin. All culture reagents were purchased from Sigma (St. Louis, MO, USA). The Caco-2 cells were originally obtained from DSMZ (Braunschweig, Germany, no: ACC 169), differentiated by serial passaging, and selected for high permissiveness to virus infection. SARS-CoV-2 was propagated in Caco-2 cells in MEM supplemented with 1% FCS, 100 IU/mL of penicillin, 100 g/mL of streptomycin, 2% glutamine, 3% amphotericin B, and 0.2% primocin (InvivoGen, San Diego, CA, USA). Inoculated cells were maintained at 37 °C, under 5% CO_2_ until complete cytopathic effect (CPE) formation. All cell culture work involving infectious SARS-CoV-2 was performed under biosafety level 3 (BSL-3) conditions. Sample inactivation for further processing was performed with methods previously evaluated [12].

### 2.3. RNA Isolation and Confirmatory RT-qPCR

Passage 0 (P0) cell culture supernatants from samples positive in the viral outgrowth assay were subjected to RNA isolation using the QIAamp 96 Virus QIAcube HT Kit on the QIAcube HT system (Qiagen, Hilden, Germany). Prior to full-genome sequencing, RNA was subjected to multiple RT-qPCR using RdRP_SARSr-F2 (5′-GTGARATGGTCATGTGTGGCGG-3′), RdRP_SARSr-R1 (5′-CARATGTTAAASACACTATTAGCATA-3′), RdRP_SARSr-P2 (5′-6-Fam-CAGGTGGAACCTCATCAGGAGATGC-BHQ1-3′) [13]. RNAseP (RPP30) was used in multiplex to monitor the input of human nucleic acids (RPP30-F: 5′-AGATTTGGACCTGCGAGCG-3′; RPP30-R: 5′- GAGCGGCTGTCTCCACAAGT-3′; RPP30-P: 5′- Cy5-TTCTGACCTGAAGGCTCTGCGCG-BHQ3-3′) [14]. 

### 2.4. NGS Sequencing of SARS-CoV-2 Genomes

NGS sequencing was performed as described previously [15]. For samples with a high viral load as determined by quantitative real-time PCR a library was prepared using up to 100 ng RNA with the KAPA RNA Hyper Prep kit (Roche Molecular Diagnostics, Basel, Switzerland) according to manufacturer’s instructions. DNA libraries were measured by Qubit dsDNA HS Assay kit (Thermo Fisher Scientific, Karlsruhe, Germany), pooled at equimolar ratios and sequenced using the Illumina MiSeq and NextSeq platforms (Illumina, San Diego, California, U.S.). For samples with a lower viral load we used a PCR amplicon-based sequencing approach. We used random hexamers and the SuperScript III Reverse Transcriptase kit (Invitrogen, Karlsruhe, Germany) according to manufacturer’s instructions and amplified by using the primer sets (V1) published by the Artic Network (https://github.com/artic-network/artic-ncov2019, access date 01/2021). A 25 μL PCR master mix was set up by using the Q5 High-Fidelity DNA Polymerase kit (New England Biolabs, Ipswich, USA) with 5 μL 5 × Q5 Reaction Buffer, 13.15 μL RNase-free water, 0.5 µL 10 mM dNTPs, 3.6 μL of either 10 μM primer pool 1 or 2, 2.5 μL cDNA and 0.25 μL Q5 High-Fidelity DNA Polymerase. PCR was carried out by using a thermocycling protocol with initial denaturation at 98 °C for 30 sec, followed by 35 cycles of 98 °C for 15 s, 65 °C for 5 min, followed by a final 2-min extension step at 72 °C. PCR products were pooled and purified using KAPA Pure Beads (Roche Molecular Diagnostics, Basel, Switzerland) according to manufacturer’s instructions.

For DNA library preparation of purified PCR amplicons, we used up to 5 ng DNA for the KAPA Frag Kit and the KAPA Hyper Prep Kit (Roche Molecular Diagnostics, Basel, Switzerland) according to manufacturer’s instructions. Sequencing was performed using the Illumina NextSeq platform (Illumina, San Diego, CA, USA).

### 2.5. Bioinformatics

**Figure 1:**
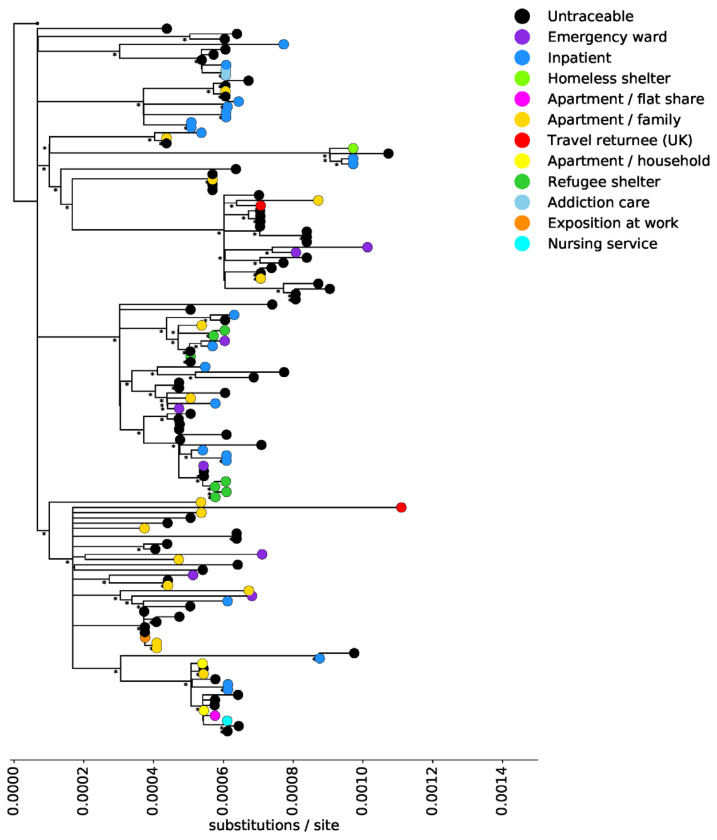
Phylogenetic tree of SARS-CoV-2 sequences: Maximum likelihood tree showing the phylogenetic relationship among the sequences generated in this study. Tips are colored by infection route. Nodes with bootstrap support less than 20 are collapsed or marked with an asterisk if bootstrap support is above 70. The tree was inferred with IQTree using a GTR substitution model and 1000 ultrafast bootstrap replicates, and is rooted with the Wuhan-Hu-1 sequence (GenBank accession no MN908947.2).

Reads were trimmed using AdapterRemoval (version 2.3.0) and aligned to the Wuhan-Hu-1 (GenBank Accession no MN908947.2) reference sequence using bowtie2 (version 2.4.1). The consensus was called using bcftools (version 1.9), requiring a coverage of at least three reads per position. Lineages were assigned using Pangolin (https://github.com/hCoV-2019/pangolin, version 2.2.2, access date 02/2021). The tree in Figure 1 was inferred from an alignment generated in MAFFT (version 7.471), using IQTree (version 2.0.3) with a GTR substitution models and 1000 ultrafast bootstrap replicates. All sequences are available on GISAID (www.gisaid.org, access date 03/2021), under accession numbers EPI_ISL_1138599- EPI_ISL_1138734.

## 3. Results

Between the end of October and the end of December 2020 (weeks 44–53), 323 positive samples from patients with SARS-CoV-2 infections were acquired by the Public Health Department of the City of Frankfurt am Main and subjected to viral cultivation assays. We found that 110 samples were positive in the viral outgrowth assay (CPE) (Table S1). Cell culture supernatants from passage 0 virus were processed for RNA sequencing while 104 yielded genome coverage > 95%. In addition, 52 samples were collected and provided by the Central Emergency Department at the University Hospital Frankfurt. Of these samples, 30 yielded genome coverage > 95%.

Nasopharyngeal swabs were taken from patients that are routinely tested using point of care PCR cartridge testing systems (GeneXpert^®^, Cepheid Inc., Sunnyvale, CA, USA) prior to inpatient admission. Two cases were associated with travel returnees from the United Kingdom. The latter samples were taken in culture, but the original swab material was subjected to sequencing. In total, we recovered 136 genomes with at least 95% genome coverage. The generated sequences represent 93% of the sequences from Hesse currently (15/2/2021) in GISAID from the period from 26 October to 29 December 2020. A phylogenetic tree showing the 136 high-coverage sequences generated in this study is shown in Figure 1. Seventy-three cases were not traceable, and the others could be assigned to a hospital (22), apartment/ family (16), emergency ward (8), refugee shelter (7), household (2), addiction care (2), travel returnee from the UK (2), shared flat (1) homeless shelter (1), exposure at work (1), and a home-care service (1), prior to or during hospitalization.

**Figure 2:**
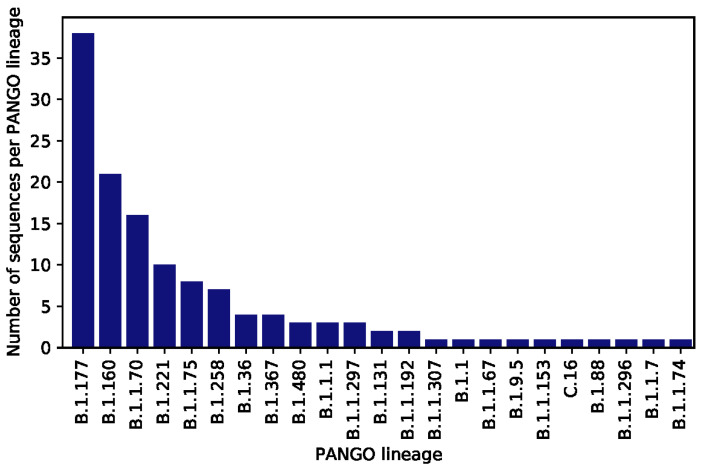
SARS-CoV-2 lineage distributions: Absolute number of sequences obtained within this study with the indicated pangolin classification.

**Figure 3:**
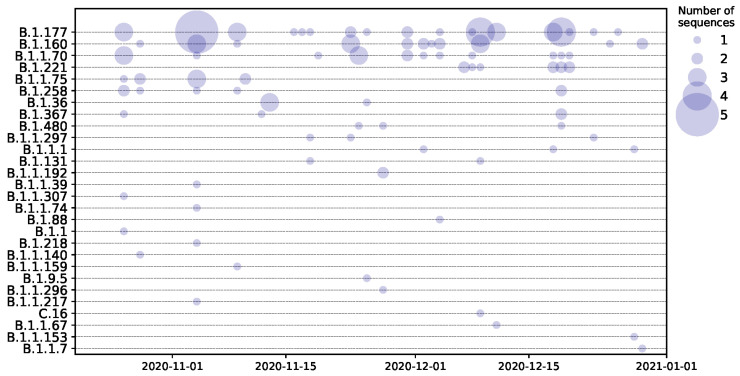
SARS-CoV-2 lineages over time: The type and number of sequences circulating per lineage over time. The size of the dot corresponds to the number of sequences represented. Lineages are ordered top to bottom according to the number of sequences assigned to a particular lineage.

Sequencing revealed 22 possible clusters involving identical sequences from an addiction care facility (one cluster), inpatients (three clusters), apartment/family and untraceable (five clusters), refugee shelter (one cluster), untraceable and refugee shelter (one cluster), untraceable and emergency ward (one cluster), untraceable and work (one cluster), apartment/family (one cluster), and untraceable and nursing service (one cluster). Sequences from patients seen in the emergency ward and inpatients are distributed throughout the tree, reflecting the diversity of viruses circulating within the catchment area of the hospital. Sequences were assigned to 28 lineages using Pangolin (Figure 2 and Figure 3). The most frequently found lineages were B.1.177 (38 sequences), B.1.160 (21 sequences), and B.1.1.70 (16 sequences), all three of which are common in Europe (Figure 2). These three lineages were found throughout the study period (Figure 3). Six lineages (B.1.1.192, B.1.1.307, B.1.1.296, B.1.1.67, C.16, B.1.1.217), have not been observed in Germany before the 15st of February 2021: lineage B.1.1.192, mainly found in the US; lineage B.1.1.307, found predominately in the UK; lineage B.1.1.296, mainly circulating in the UK, US, and UAE; lineage B.1.1.67, mainly in the UK and Russia; lineage C.16 found in Portugal, UK, Switzerland and France; and lineage B.1.1.217, mainly found in the UK. None of the sequences assigned to those lineages were found in individuals with known recent travel history. 

Four sequences falling into clade B.1.211 have an identical 41 bp deletion in the 3′ untranslated region (Table 1). Eighteen sequences have a deletion at position 69/70 (Δ69/70) in S. One of these sequences can be assigned to lineage B.1.1.7, 12 to lineage B.1.1.70, and five to lineage B.1.258. The sequences in the latter lineage also have the N439K substitution in S. The B.1.1.7 sequence was recovered from a person with travel history to the United Kingdom, initially tested positive for SARS-CoV-2 sampled on 29 December 2020 for sequencing. Another sample tested positive at the emergency ward on 27 December 2020, yielding a partial sequence (< 95% genome coverage, data not shown) which could also be assigned to the B.1.1.7 lineage on the basis of its substitutions. This represents the first sequenced importation of a virus from the B.1.1.7 lineage documented in the state of Hesse. One of the sequences falling into clade B.1.258 has an additional deletion of three base pairs at amino acid position 58 in ORF8. Two sequences have a substitution at position 501 of the S protein from Asparagine to Tyrosine. One falls into clade B.1.1.7 while the other is assigned to lineage B.1.1.70. 

Four monophyletic sequences assigned to lineage B.1.36 have a deletion at amino acid position 210 in S. One sequence has a deletion of four amino acids (amino acid positions 141-144) in S. Position 144 is also deleted in lineage B.1.1.7. Three monophyletic sequences in lineage B.1.160 have a four base pair deletion in ORF3a which leads to a truncation of the gene at amino acid position 259. The same deletion is present in a sequence in lineage B.1.1.67. In an additional sequence also falling into clade B.1.160, ORF3a is truncated in the same location, but by a one base pair deletion. Finally, one sequence has a nine base pair deletion in ORF1ab (amino acid positions 141-143) and a seven bp deletion in ORF7a, which truncates ORF7a at amino acid position 108.

## 4. Discussion

RNA viruses, such as the human immunodeficiency virus (HIV), hepatitis C virus or influenza, are prone to high error rates caused by their RNA polymerases. In comparison the, sequencing data from SARS-CoV-2 suggests a significantly lower mutation rate, which might be a result of the proofreading activity of the viral nsp14 [16]. SARS-CoV-2 accumulates approximately two mutations per month in its genome, which is considerably less than influenza and HIV [17]. However, a high incidence and uncontrolled spread increase the likelihood of mutation. In addition, residue mutation rates seem to be dynamic and differ for each residue such that the Spike protein shows higher mutation frequency than Envelope protein [18]. Geographical and temporal differences such as seasonal changes, country-specific factors, demography, cultural and social interventions, and surveillance mechanisms can condition the emergence of different SARS-CoV-2 variants. 

In this study we recovered 136 sequences of SARS-CoV-2 circulating in Frankfurt am Main from the end of October to the end of December 2020. We observed 28 circulating lineages, six of which have not previously been found in Germany. Of those, none were found in patients with a known recent travel history. The sequences generated in this study only represent a small fraction of the total number of people that tested positive in the city during the study period. The lineage diversity we describe therefore represents a lower bound estimate of the total diversity that may have been circulating between the end of October and the end of December. 

The possibly increased transmissibility or antigenic variability of the emerging variants may lead to an increased number of cases, hospitalizations, and deaths. For B.1.1.7, epidemiological and phylodynamic modelling suggest a 43%-90% increase in the reproduction number compared to previously circulating variants, providing evidence of higher transmissibility [19]. Recent data additionally suggest that infections with B.1.1.7 may be associated with increased case fatality rates [20].

During this study, we found one sequence with the N501Y substitution in the S protein that is not associated with a variant of concern lineage, but with lineage B.1.1.70. Of the 1917 sequences in GISAID currently (15/2/2021) assigned to clade B.1.1.70, ten have Δ69/70, seven of them from Germany, sampled since late December, to which we have added a further nine sequences. We found the N501Y substitution in 610 sequences in clade B.1.1.70 (one from Germany, the majority from Wales). Within the same lineage, we thus see the independent acquisition of two mutations found in variants of concern. This observation further supports the evolutionary convergence of N501Y as an initial key event in different viral clades [21]. The typing PCRs commonly used for the identification of the B.1.1.7 and B.1.351 variants target the Δ69/70 deletion and the N501Y substitution in S. We show that nonvariant of concern sequences may also harbor the N501Y substitution, and may be common in Germany, suggesting that a positive typing PCR only for N501Y should be followed up by additional full-genome sequencing.

We found no evidence for a circulating variant harboring the E484K mutation during this study period, which is present in the B.1.351 (first identified in South Africa), P.1, and P.2 (both originating from Brazil lineages). Immune evasion against monoclonal antibody preparations and reduced neutralization capacity against vaccine-elicited and convalescence sera have been described for variants carrying the E484K substitution, making these lineages of particular concern [22,23,24,25].

Monitoring fluctuations in transmission rates and identifying emerging variants that impact spread are pivotal to disease control. Together with the recent introduction of the variants of concern B.1.1.7 and B.1.351 into Germany, these findings highlight the urgent need for increased and continuous surveillance using complete genome sequencing to monitor the virus diversity currently circulating in Germany.

## Figures and Tables

**Table 1:** Deletion and/or amino acid substitutions identified in the recovered SARS-CoV-2 genomes.

Deletion/Mutation	Number of Sequences	PANGO Lineage	Sequence ID
9 bp deletion in ORF1ab (amino acid position 141–143), 7 bp deletion in ORF7a which truncates ORF7a at amino acid position 108	1	B.1.160	ChVir21561
Deletion of amino acid position 69/70 in S	17	B.1.1.70, B.1.258	ChVir21551, ChVir21580, ChVir21582, ChVir21585, ChVir21586, ChVir21588, ChVir21589, ChVir21591, ChVir21596, ChVir21597, ChVir21598, ChVir21606, ChVir21609, ChVir21618, ChVir21619, ChVir21621, ChVir21626
Deletion of amino acid positions 141-144 in S	1	B.1.1.153	ChVir22027
Deletion of amino acid position 210 in S	4	B.1.36	ChVir21555, ChVir21563, ChVir21571, ChVir21603
N501Y substitution in S	1	B.1.1.70	ChVir21997
4 bp deletion in ORF3a, which truncates ORF3a at amino acid position 259	3	B.1.160, B.1.1.67	ChVir21565, ChVir21625, ChVir21632, ChVir22011
1 bp deletion in ORF3a, which truncates ORF3a at amino acid position 259	1	B.1.160	ChVir21550
Deletion of amino acid position 58 in ORF8	1	B.1.258	ChVir21586
12 bp deletion in 3p UTR	1	B.1.258	ChVir21502
41 bp deletion in the 3’ UTR	4	B.1.221	ChVir21994, ChVir21995, ChVir21996, ChVir22006
Substitutions typical of B.1.1.7, including a deletion of amino acid position 69/70 and N501Y in S	1	B.1.1.7	ChVir22031

## Data Availability

All sequences are available on GISAID (www.gisaid.org), under accession numbers EPI_ISL_1138599- EPI_ISL_1138734.

## Supplementary Materials

The following are available online at https://www.mdpi.com/article/10.3390/microorganisms9040748/s1, Table S1: Patient data: Characteristics of clinical samples by source, cycle threshold (Ct) values, date of collection, PANGO lineage, genome coverage, and sequence features. CPE positive indicates successfully outgrowth virus cell culture.
